# Catheter ablation of premature ventricular contractions using an alternative vascular access and remote magnetic navigation (ARM) approach

**DOI:** 10.1093/europace/euag136

**Published:** 2026-06-03

**Authors:** Sabine Ernst, Rui Shi, Bruce Barton, Ghada Mustafa, Jack R Griffiths

**Affiliations:** Department of Cardiology, Royal Brompton Hospital, Guy’s and St.Thomas’ NHS Foundation Trust, Sydney Street, London SW3 6NP, UK; National Heart and Lung Institute, Imperial College, London, UK; Department of Cardiology, Royal Brompton Hospital, Guy’s and St.Thomas’ NHS Foundation Trust, Sydney Street, London SW3 6NP, UK; Department of Cardiology, Royal Brompton Hospital, Guy’s and St.Thomas’ NHS Foundation Trust, Sydney Street, London SW3 6NP, UK; Department of Cardiology, Royal Brompton Hospital, Guy’s and St.Thomas’ NHS Foundation Trust, Sydney Street, London SW3 6NP, UK; Department of Cardiology, Royal Brompton Hospital, Guy’s and St.Thomas’ NHS Foundation Trust, Sydney Street, London SW3 6NP, UK

**Keywords:** Premature ventricular contractions, ECG imaging, Remote magnetic navigation, Peripheral vascular access, Radiofrequency catheter ablation

## Introduction

Catheter ablation of premature ventricular contractions (PVCs) is nowadays state-of-the-art treatment in patients with high PVC burden or when highly symptomatic.^1^ These procedures, however, have traditionally been conducted via femoral vascular access(es). We recently reported on two patients with interrupted or occluded femoral vessels, who underwent successful catheter ablation of supraventricular arrhythmias via an alternative vascular access and remote magnetic (ARM) approach.^2^ In this article, we present an initial case series of patients with PVCs using this novel approach.

## Methods

This is a single-centre, retrospective analysis enrolling patients undergoing ablation for PVC at Royal Brompton Hospital, London. In 9/11 procedures, the non-invasive ECG-imaging platform Vivo (Catheter Precision)^3^ was used to non-invasively predict PVC origin while the patient was still on the ward. All patients were studied in a fasted state, and after informed consent was obtained. Anaesthesia ranged from local anaesthetic to deep sedation using a laryngeal mask delivered by a cardiac anaesthetist (as per our institutional standard).

Vascular access was obtained under ultrasound guidance following the same technical recommendations as for PICC line placement.^4^ Endocardial mapping and ablation were performed using a single-catheter approach with either the 8F Navistar RMT (Biosense Webster, *n* = 10) or 7F MagnoFlush (MedFact, *n* = 1) catheters. All procedures were performed using either the CARTO RMT (*n* = 10) or the ENSITE 3D mapping system.

Continuous variables were either reported as mean ± standard deviation (if normally distributed) or as median (interquartile range).

## Results

Ten consecutive patients (six women, mean age 50 years) with documented PVCs underwent invasive electrophysiology studies using the ARM approach. One patient required a repeat ablation procedure, and both procedures were performed using the ARM approach. Patients had a structurally normal heart (*n* = 8) or congenital heart disease (*n* = 2: ASD repair, AVSD repair). The magnetic ablation catheter was initially advanced into the right ventricle from the left brachial (*n* = 5), left basilic (*n* = 3) or right basilic veins (*n* = 3), respectively. In 5 procedures, radial (*n* = 4) or brachial (*n* = 1) arterial access was also obtained to allow a retrograde approach in the left ventricle (LV) and aortic cusps.

**Figure 1:**
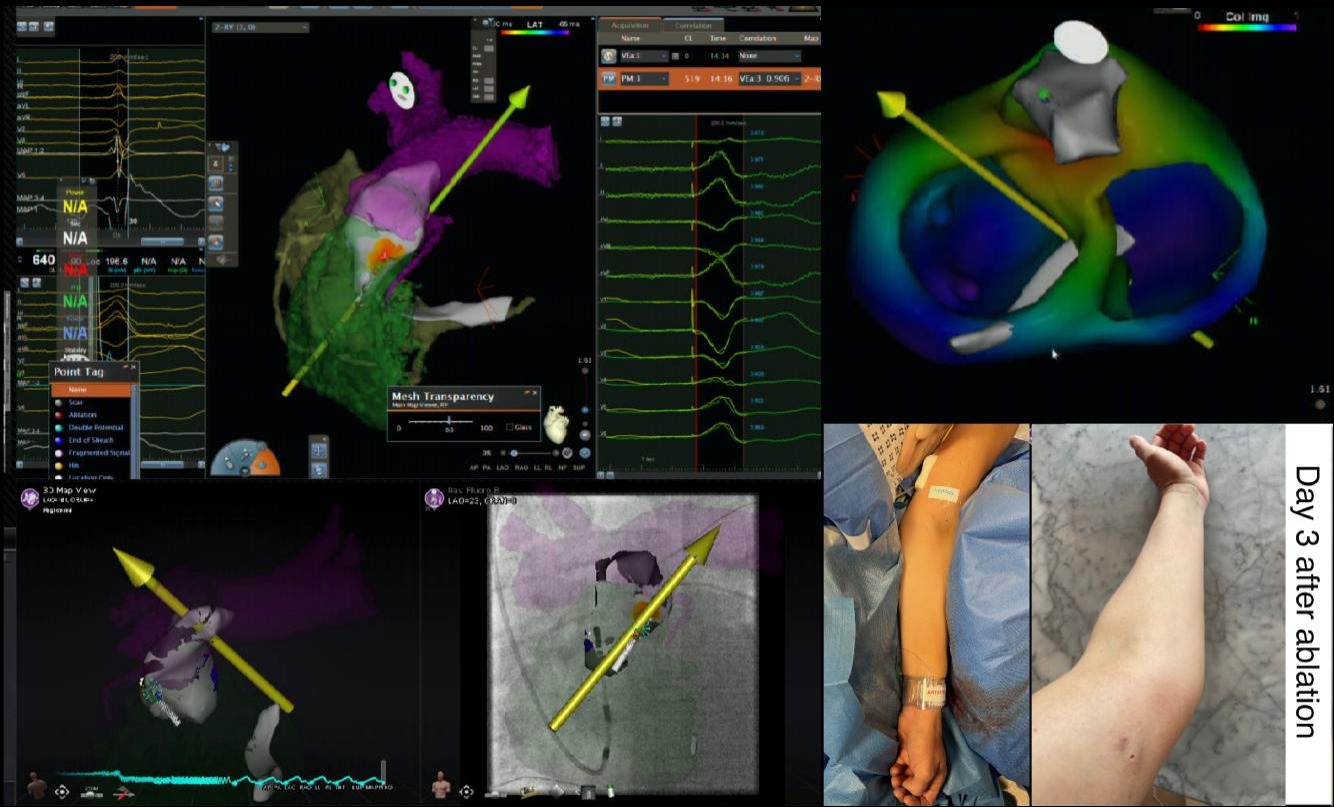
Composite figure demonstrating the access sites from the left basilic vein and left radial artery. The arrow depicts the direction of the external magnetic field that allows navigating the soft ablation catheter from the arm to the right ventricular outflow tract (note the catheter arriving via the SVC on the fluoro image). The right upper figure depicts the non-invasive 3D mapping information in a tilted postero-anterior projection with the septal aspect of the right ventricular outflow tract being the earliest (red colour). The two right lower pictures illustrate the access sites immediately post and 3 days after the procedure.

In 11 procedures, PVC origin was localized to the right ventricular outflow tract, and in 5 patients, the adjacent aortic cusp and/or distal coronary sinus was mapped as well (*Figure 1*). In 9/11 procedures, the non-invasive 3D mapping system was used and correctly predicted the site of origin of the PVC in all cases. Conventional mapping techniques, including pace mapping, earliest local bipolar activation, and unipolar qs signal recordings, were used for confirmation of PVC origin.^5^ Radiofrequency, applied at 50–70 Watts for a median duration of 8.9 min (range: 4.9–11.9 min), effectively eliminated all PVCs acutely. Overall median procedure time was 105 min (range: 83–160 min). Fluoroscopy usage lasted a median of 20 s (range: 10–25 s) and resulted in a radiation dose of 3.9 cGycm2 (range: 3–10.2 cGycm2). No acute complications were noted, except for temporary bruising at the puncture site (*Figure 1*). 12-month follow-up demonstrated elimination of any significant PVC burden in 10/11 patients, with one patient with an intramural origin ultimately undergoing re-ablation using pulsed field energy.

## Discussion

To the best of our knowledge, this study presents the initial experience of using peripheral vascular access from the arms for magnetic navigation-guided catheter ablation of ventricular arrhythmias.

### Safety and efficacy

Acute procedural and follow-up results of our ARM approach suggest excellent safety and efficacy. Notably, patients can ambulate immediately post-procedure, alleviating the need for bed rest and enhancing patient comfort and satisfaction. This may make it an attractive approach in the novel era of ambulatory electrophysiologic procedures aiming at same-day discharge.^6^

### Single catheter approach plus non-invasive 3D mapping

Single catheter PVC ablation has been promoted recently and demonstrated excellent clinical outcomes.^7^ We adopted this single catheter approach and added the use of non-invasive 3D mapping on the ward in most of our cases. It facilitated the procedure, and similar procedure parameters were achieved as compared to reports using a femoral approach.^3,8^ However, if needed additional diagnostic catheters can be added as there are typically several arm vessels amenable for alternative access (such as brachial or cephalic veins).

### Comparison to contemporary PVC ablation via femoral access

When comparing our results to contemporary publications on PVC ablation procedures, parameters such as procedure duration are indeed comparable, but our low overall fluoroscopy exposure is notable.^8,9,10^ As the soft magnetic ablation catheter can be advanced without the fear of perforation, following the icon of the catheter tip on the 3D mapping system is sufficient to navigate it into the heart. The ability to directly steer the magnetic tip of a very flexible catheter, rather than a stiff catheter with a pull-wire mechanism, enhances the accessibility of all areas of the cardiac anatomy (as demonstrated here for the right ventricular outflow tract and the adjacent aortic cusps). Lastly, the picture-in-picture display of the 3D map on the fluoroscopy reference image with real-time depiction of the catheter icon also aids in reducing fluoroscopy exposure (*Figure 1*).

Nevertheless, it is essential to emphasize that our findings are preliminary, originating from a single centre, and are limited by a small sample size.

## Future outlook

Looking ahead, advancements in catheter design, such as the development of a dedicated slimmer ARM catheter (e.g. 6 French), ideally also offering a pulsed field ablation option, would be optimal.

## Conclusion

We report on an alternative access remote magnetic (ARM) approach via the vasculature of the arm for single catheter ablation of PVCs with excellent safety and efficacy. Procedure parameters were comparable to procedures via conventional femoral access but excelled at very low radiation exposure.

## Data Availability

Data is available on request to the corresponding author.
